# Carrier Variety Used in Immobilization of His_6_-OPH Extends Its Application Areas

**DOI:** 10.3390/polym15030591

**Published:** 2023-01-24

**Authors:** Elena Efremenko, Ilya Lyagin, Aysel Aslanli, Nikolay Stepanov, Olga Maslova, Olga Senko

**Affiliations:** Faculty of Chemistry, Lomonosov Moscow State University, Lenin Hills 1/3, Moscow 119991, Russia

**Keywords:** organophosphorus hydrolase, hexa-histidine tag, immobilization, stabilization, biocatalysis, *N*-acyl homoserine lactones, organophosphorus compounds, mycotoxins

## Abstract

Organophosphorus hydrolase, containing a genetically introduced hexahistidine sequence (His_6_-OPH), attracts the attention of researchers by its promiscuous activity in hydrolytic reactions with various substrates, such as organophosphorus pesticides and chemical warfare agents, mycotoxins, and *N*-acyl homoserine lactones. The application of various carrier materials (metal-organic frameworks, polypeptides, bacterial cellulose, polyhydroxybutyrate, succinylated gelatin, etc.) for the immobilization and stabilization of His_6_-OPH by various methods, enables creation of biocatalysts with various properties and potential uses, in particular, as antidotes, recognition elements of biosensors, in fibers with chemical and biological protection, dressings with antimicrobial properties, highly porous sorbents for the degradation of toxicants, including in flow systems, etc. The use of computer modeling methods in the development of immobilized His_6_-OPH samples provides in silico prediction of emerging interactions between the enzyme and immobilizing polymer, which may have negative effects on the catalytic properties of the enzyme, and selection of the best options for experiments in vitro and in vivo. This review is aimed at analysis of known developments with immobilized His_6_-OPH, which allows to recognize existing recent trends in this field of research, as well as to identify the reasons limiting the use of a number of polymer molecules for the immobilization of this enzyme.

## 1. Introduction

Interest in the enzyme aryldialkylphosphatase (EC 3.1.8.1)—which is now known in the scientific literature under two names that are actively used in various studies, organophosphorus hydrolase (OPH) and phosphotriesterase (PTE)—increases due to a number of objective reasons. On the one hand, among these reasons are problems associated with the widespread use of organophosphorus compounds (OPCs) as agricultural pesticides [1], their toxic properties, and their ability to accumulate in various biological objects, environmental objects (aquatic environments and soils), as well as organs and tissues [2]. On the other hand, this is dictated by the need to degrade these substances in the most favorable conditions for ecosystems and their components, including animals and humans, and to carry out bioremediation measures aimed at reducing pollution of environmental objects (soil, water, and air), food, and agricultural raw materials by various OPCs [3]. In addition, there is a demand for highly effective antidotes in the event of serious cases of human intoxication with such compounds [2], which may be the result of their penetration into the human body through oral, inhalation, and/or transdermal routes.

Today, the possibility of using enzymes as catalysts for the destruction of OPCs is characterized by a fairly high action efficiency and a wide range of different substrates—which, as it turns out, may include not only the OPCs and not only pesticides, but also chemical warfare agents (CWAs) as well as products of their chemical and biocatalytic degradation [3,4,5,6].

**Figure 1:**
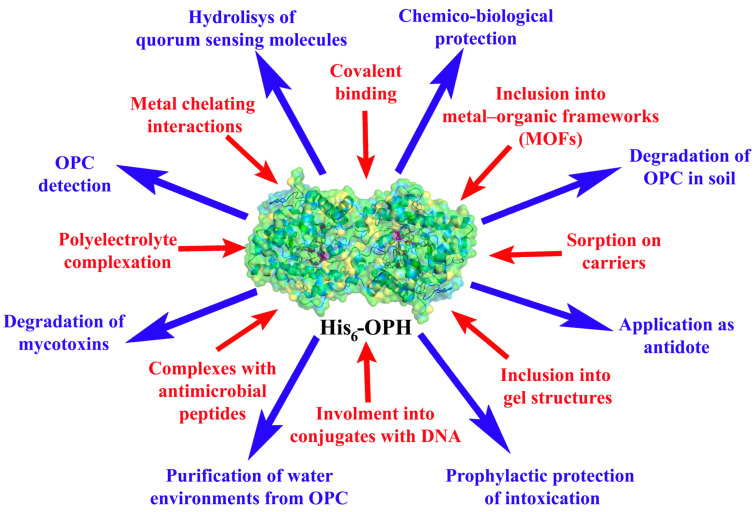
Various methods of immobilization (red) and applications (blue) of hexa-histidine-containing OPH.

Among the main advantages of using enzymes for the decomposition of OPCs, especially in natural objects, are their high level of catalytic activity, chemical and stereoselectivity, minimal amounts of byproducts, and compatibility with different biological systems [7]. The latter is extremely important when the enzyme is used as a part of a hydrolytic antidote or a long-circulating preventive nanoscavenger acting against the OPCs in vivo [8,9] (Figure 1). In addition, the enzymatic hydrolytic degradation of OPCs can be used for the biosensing determination of these substances [10,11]. 

It should be noted that the search for new sources of OPH, the genetic modification of which is carried out through the use of various plasmids encoding the synthesis of an enzyme with a polyhistidine sequence mainly at the *N*-terminus of a protein molecule, is constantly being conducted in different laboratories around the world [5,12,13,14]. A study of the properties of such genetically modified OPH variants has shown that the presence of a hexahistidine tag in the enzyme molecule (His_6_-OPH) gives it new properties: stability of action in the presence of various surfactants [15], action at increased temperatures, and changed pH of the medium [16]. The improved biocatalytic characteristics and altered stereospecificity of the catalytic action were also revealed for the His_6_-OPH as compared to OPH [17]. The latter property can be achieved by combining the genetic introduction of a polyhistidine sequence into the enzyme molecule with site-directed mutations in the active center of the enzyme and in the amino acid residues around it or in the substrate-binding domain [11,18].

For a number of enzymes, the term PTE-like lactonases (PLLs) has been introduced, since they had a fairly high homology with PTE/OPH but low activity in OPC hydrolysis reactions compared to OPH. These enzymes had greater hydrolytic activity against *N*-acyl homoserine lactones (AHLs), which are signaling molecules of the quorum of Gram-negative bacteria. Verification of similar lactonase activity in His_6_-OPH led to the conclusion that this enzyme may be of practical interest not only for solving the issues of destruction and detection of OPCs but also for serving as the basis for such a process as Quorum Quenching (QQ), since the lactonase activity of His_6_-OPH in reactions with different AHLs was comparable with that of PLLs [19] (Figure 1).

The analysis of the substrate specificity of the action of His_6_-OPH in reactions with lactone-containing compounds led to the idea of using this enzyme for the destruction of mycotoxins, in particular, patulin and zearalenone, which are lactones [20]. The success of such catalytic reactions made it possible to consider His_6_-OPH as an enzyme that hydrolyzes a number of mycotoxins that form serious problems related to the quality of agricultural raw materials and animal products. Today, new results with His_6_-OPH in mycotoxin degradation reactions suggest the promiscuous activity of this enzyme [21], which further increases the scientific and practical interest in it (Figure 1).

Today, the main task of researchers, who are engaged in conducting research and creating new developments based on His_6_-OPH, is to create stable and active forms that could show their high functionality in various environments and reactions with the above substrates (OPCs, AHLs, and mycotoxins). It should be noted that earlier, similar studies obtaining a variety of stabilized forms of hexa-histidine-containing OPH were carried out, and there are separate examples of the analysis of such developments [2,11,13]. However, these publications contain information reflecting only the results of the study of stabilized His_6_-OPH participating in the reactions of OPC destruction, and do not affect new data on the study of the properties of this enzyme in a stabilized form in reactions with AHLs and mycotoxins. In this regard, when referring to such an analysis in this review and making it the purpose of this publication, exceptional attention was paid to the survey of new, stabilized forms of hexa-histidine-containing OPH and the methods used for this, the results of which were published only within the last 5 years. At the same time, the results of the application of various methods of His_6_-OPH immobilization as a result of covalent and non-covalent interactions of the enzyme with its stabilizing polymer effectors were compared.

## 2. Stabilization of Hexa-Histidine-Containing OPH for OPC Hydrolysis

When discussing the possibilities of using various approaches to stabilize hexahistidine-containing OPH, it should be noted, first of all, the expediency of using carriers for immobilized metal ion affinity chromatography is intended for the interaction through which His_6_-tag is generically introduced into the enzyme molecule. It is on this interaction of the affine sequence and metals in the composition of chelating groups (which, as a rule, modify various polymer carriers (agarose, cryogels of synthetic and natural polymers, etc.)) is based that fast and highly effective immobilization, and then isolation and purification of the enzyme itself occurs. The emerging coordination bonds between metal atoms in the carrier and nitrogen atoms in the imidazole rings of the polyhistidine sequence in the protein molecule make it possible to anchor the enzyme on the carrier quite firmly [2,22,23] and use it for a long time, in such an immobilized form, for the destruction of OPCs (Table 1, [23,24,25,26,27,28,29,30,31,32,33,34,35,36,37,38,39,40,41,42,43,44,45,46,47]). Of particular interest are such immobilization variants for the stabilization and repeated use of the enzyme in the degradation of OPC-polluting water systems [22,23]. In fact, another variant of carriers that can be used for the ion-chelating immobilization of His_6_-OPH is metal–organic frameworks (MOFs), which are usually used as porous functional materials [48]. They are formed through the self-assembly of organic ligands containing oxygen or nitrogen atoms and metal atoms (or metal clusters) as the inorganic part of MOFs. By varying organic ligands and metals, it is possible to control the pore size of materials, the specificity and functional modification of their surface area, and their interaction with various proteins. Owing to these capabilities, the MOFs are widely used in the study of catalytic processes and biosensors, generally. MOFs used for the immobilization of enzymes usually provide significantly improved stability and recyclability of biocatalysis in diverse applications [49,50,51,52,53,54].

The interest in such carriers is additionally motivated by the possible detoxifying effects of self-MOFs in relation to toxins, including OPCs (pesticides and CWA) [52,53,54]. Since then, MOFs as supports look very attractive for the obtainment and use of immobilized forms of His_6_-OPH [24,25,26,27,28,29].

Combinations of MOFs with enzymes catalyzing the hydrolysis of OPCs are promising since they allow for the combination of the potential of chemical and biological catalysts. An example of such a combination obtained on the basis of His_6_-OPH is the development of a conjugate of this enzyme with UiO-66-NH_2_-containing MOFs [26]. This variant of the enzyme combination with MOFs guarantees the production of a catalyst, which is characterized by several improved properties at once (activity, stability, substrate specificity of action), allowing for the use of such an immobilized enzyme both for the decomposition of methyl parathion and for its detection in fairly low concentrations (10 ng/mL) and, at the same time, in a fairly wide range of concentrations (10–10^6^ ng/mL) [26].

The use of MOF modified by Terbium-BTC, where BTC is 1,2,4-benzenetricarboxylic acid, for the encapsulation of His-tagged organophosphorus hydrolase (OPH^6His^) allowed the authors of the development to improve the stability of the enzyme and increase the activity of the resulting biocatalyst by 30% in comparison with the original enzyme [27]. The sample of the modified enzyme was obtained by mixing terbium nitrate pentahydrate (Tb(NO_3_)_3_ × 5H_2_O) and an aqueous solution of the enzyme and BTC in ethanol. It was successfully applied for providing a highly sensitive determination of methyl parathion in grapes and tomatoes with a low enough limit of detection (2.6 nM). 

An interesting solution of coordinating the immobilization of His-tagged organophosphohydrolase (OpdA) via the metal chelating interactions was realized using the yolk-shell structured Co/C@SiO_2_@Ni/C nanocomposites [28]. It was obtained through the carbonization treatment of the polyhedral ZIF-67@SiO_2_ composite. The stabilization of the structure of the carbonized nanocomposite was completed through the creation of SiO_2_-layer protection. Further, the Ni-nanoparticles were introduced to the surface of the formed structure and further enzymes were successfully immobilized. This example of the combined variant of the chemo-enzymatic nanocatalyst was capable of hydrolyzing OPCs and detecting methyl-parathion with a low enough limit of detection (300 nM). Comparisons of the activity and stability of the immobilized biocatalyst against the initial enzyme demonstrated their improvement.

Recently developed and reviewed methods of obtaining new hybrid nanostructures, named “nanoflowers” [55,56,57], were applied for the immobilization of His-tagged PTE [29]. Usually, chlorides or phosphates of metals are used in the mixture with proteins for the creation of organic–inorganic structures of nanoflowers. In this investigation, the combination of the concurrent introduction of both Co and Mn provided the best results with the enzyme.

The use of a carrier enzyme (hollow-structured nanoparticles, Au-TiO_2_) for immobilization, which, due to its composition, provides the possibility of the photocatalytic decomposition of OPCs, makes it possible to obtain a material with new functions combining chemical and biological catalysis. This makes the process of the degradation of toxins more resistant to the variation in external factors, and the material itself, with the synergistic effect of its constituent components, allows for more long-term use [30]. It should be noted that the immobilization itself leads to a noticeable decrease in the hydrolytic activity of the enzyme, but the possibility of photocatalysis compensates for this decrease during the decomposition of methyl parathion.

The use of hollow-structured nanoparticles, Au-TiO_2_, as a carrier for enzyme immobilization (which, due to its composition, provides the possibility of photocatalytic decomposition of OPCs) makes it possible to obtain a material with new functions combining chemical and biological catalysis, which makes the process of degradation of organophosphorus toxins more resistant to the variations in external factors. The material itself, with the synergistic effect of its constituent components, allows for more long-term usage [30]. It should be noted that the immobilization itself leads to a noticeable decrease in the hydrolytic activity of the enzyme, but the opportunity of photocatalysis compensates for this decrease during the decomposition of methyl parathion.

Polyhistidine-containing OPH was immobilized on a carrier known as Fuller’s Earth, which, in addition to hydrated aluminum-magnesium silicate, contained various metal ions (Mg^2+^ and Ca^2^) as well as montmorillonite, kaolinite, and attapulgite/bentonite [32]. The carrier used is known for its high sorption capacity with respect to proteins as well as to various chemical agents and toxins, and this possibility provided the enzyme with the presence of a substrate in excess, simulating catalytic (non-limiting) reaction conditions.

A fundamentally different approach, based on the use of polyelectrolyte interactions of the surface of the His-tagged enzyme with variously charged surfactants, was used by researchers for the creation of various bioconjugates to improve the stability and catalytic characteristics of the enzyme [33,34,35]. At the same time, the expediency of using such coatings for the enzyme, including forming a capsule in the form of a so-called corona, was noted. Such structures made it possible to avoid the direct influence of various unfavorable factors on the enzyme itself. They simultaneously retained the possibility for the covalent modification of surfactants, the possible addition of various polymers to them, and their introduction into diverse plastics, fabric materials, and 3D printing systems, allowing the enzyme to be applied to metal surfaces as a part of the created conjugates. This creates scientific and practical foundations for the development of personal protective equipment against the toxic effects of OPCs. Additional genetic modifications of the enzyme itself—particularly due to the connection with the fusion protein (GFP) [33] before its encapsulation in the layers of surfactants—create the possibility of additional control of the effect of immobilization conditions on the properties of the main biocatalyst through the properties of the selected fusion partner (fluorescence intensity of GFP).

The traditional approach to the immobilization of His_6_-tagged OPH continued to be developed in recent years, and the cross-linking conjugation of the enzyme modified by the genetic introduction of 10 residues of phenylalanine was demonstrated with Pluronic F127 [36]. This work and another study based on the application of mesoporous silica nanoparticles coated with a zwitterionic polymer [37] were good illustrations for the successful covalent binding of the enzyme to a polymeric molecule to retain and improve its stability. The results obtained [36] revealed better catalytic activity of enzyme conjugates as compared to samples prepared by the covalent immobilization of the same enzyme into well-known, cross-linked enzyme aggregates (CLEA) by using glutaraldehyde.

An example of how His_6_-tagged OPH can be immobilized by sorption and covalent immobilization on natural carriers, varying the properties of the carriers themselves, are studies based on poly-β-cyclodextrin [38] and polyhydroxyalkanoate) (PHA) microspheres [39,40]. Interestingly, multiple modifications of these microparticles with the introduction of various spacers and fusion proteins onto their surface had virtually no effect on the fact that, in the end, the activity of the enzyme under study exceeded the same parameter known for the soluble enzyme.

The solution arrived at by the authors of the development, in which His_6_-tagged OPH was connected directly to the shells of PEGylated quantum dots (QD) through a linker, which was comprised of DNA connected to nanoparticles, looks unusual and promising [41,42]. Firstly, the DNA appeared to be a good anchor for the enzyme, since it provided the availability of its active center for catalysis and ensured its successful conformation, which contributed to the increase in the OPH catalytic constant in paraoxon hydrolysis. Secondly, the modification of DNA molecules—on the one hand by an enzyme, which can potentially be used as an antidote, and on the other hand, the possible attachment of DNA to nanoparticles, which can have targeted delivery to certain cells and tissues—can be continued in the form of the development of new nanobiosensors.

It is interesting to note that an increase in the DNA concentration relative to QD-OPH and the creation of dense composites led to an increase in the enzymatic reaction rate by 12.5 times [43]. It should be noted that, in principle, the use of DNA molecules modified for their use as bioscaffolds for the enzyme under discussion has proven to be very good in terms of an effective means of improving the catalytic characteristics of the obtained biocatalysts even in the absence of QD [44]. It turned out that, depending on how the site-specific introduction of functional groups into the enzyme molecule for its subsequent conjugation with DNA occurs, *K*_M_, *k*_cat_, and, accordingly, their ratio (*k*_cat_/*K*_M_) can be directionally changed.

The method of immobilization and stabilization of His_6_-tagged OPH, due to the formation of various polyelectrolyte complexes, turned out to be very simple in implementation and very promising according to the results of its application [8,9,44,45,46,47]. At the same time, proteins (gelatin), poly(carboxybetaine), poly(amino acids), and their copolymers with poly(ethylene glycol), turned out to be partners in such enzyme–polyelectrolyte complexes. All these developments have proved to be highly successful not only in terms of the simplicity of the formation of stable forms of the enzyme (characterized by significantly improved resistance in the manifestation of catalytic activity with a significant decrease in pH and a temperature increase) but also in terms of their possible applications—not only to solve problems of OPC destruction in aqueous media, in particular samples of contaminated soils [45], but also in the blood [8,9] and gastrointestinal tracts of animals [46]. Thus, the preparation of OPT-PIMs enzyme complexes allowed the authors to develop a sample of an enzyme included in a polyelectrolyte complex, whose microparticles can be consumed by bees (together with plant pollen particles) and catalyze the destruction of several organophosphorus pesticides while maintaining activity at pH 4.8 in the insect digestive system.

The creation of non-covalent complexes based on His_6_-OPH and the PEG_113_-PLE_50_ block copolymer [8] showed that the enzyme acquires improved catalytic characteristics and increased stability during storage compared to the original enzyme. The introduction of such non-covalent complexes in vivo showed a decrease in immune and inflammatory reactions to His_6_-OPH. The pharmacokinetic parameters of such complexes were also improved compared to the soluble enzyme, which ensured their longer circulation in the bloodstream after intravenous (iv) administration in rats. In addition, the enzyme remained bioavailable after intraperitoneal (ip), intramuscular (im), and even transbuccal (tb) administration and had the ability to protect animals from exposure to OPCs (paraoxon, VX) in vivo.

The development of similar biocatalysts, by applying a thin, ultra-hydrophilic, semi-permeable polymer gel layer poly(carboxybetaine) (PCB) to the surface of the enzyme [9], showed that the resulting gel-coating layer of the zwitterionic polymer also provides long-term circulation and minimal immunogenicity of the resulting form of the enzyme biocatalyst. The study of the properties of such an enzyme in vivo in models of different animals before or after exposure to OPCs has demonstrated the protective and antidote effectiveness of their action. In guinea pig models, a single prophylactic administration of such a biocatalyst effectively prevented the mortality of animals after repeated exposure to sarin for 1 week. The study showed an increase in catalytic characteristics and the stabilization of the enzymatic activity of His_6_-tagged OPH. The results obtained show that the profile application of such a biocatalyst can be effective in preventing the toxic effects of OPCs (paraoxon, sarin) in vivo.

It should be noted that the variants of the immobilization of His_6_-tagged OPH for the hydrolysis of OPCs described here have been developed by researchers only in recent years, but even their diversity is very wide. It should be noted that the use of the enzyme under discussion is the most well-studied precisely for the degradation of OPCs. However, the spectrum of the substrates of this enzyme is not limited to OPCs [2,19,20], and therefore, the options for its stabilization and immobilization for use in other hydrolytic reactions remain extremely interesting. A lot of parameters of biocatalytic systems can be predicted in silico: the interaction of the enzyme with novel substrates [4,6], the interaction of the enzyme microenvironment/partner with substrates [43], the binding of enzymes with individual polymers [46], etc. Prospectively, computer modeling may allow the designing of the necessary catalytic system on a turnkey basis.

## 3. His-Tagged OPH Involved in the Immobilized Antimicrobial Combinations

Since the ability of His_6_-OPH to hydrolyze lactone-containing compounds—in particular, the quorum molecules of Gram-negative bacteria—has long been evolutionarily motivated [58,59] and experimentally confirmed [19,60], the possibility of using this enzyme as an effective tool for the bacterial QQ-process is of scientific and practical interest. 

The most appropriate practical application of His_6_-OPH, in this case, seems to be in combination with antimicrobials, which can have a major suppressive effect on microbial cells; then, the enzyme becomes a tool for weakening the resistance of cells to antimicrobial effects. Since the presence of antimicrobial agents can have a negative effect on the manifestation of enzyme activity, a significant role is currently assigned to computer methods of molecular modeling [4,6,46,60,61,62,63], including the search for the most successful partners to create effective antimicrobial combinations based on His_6_-tagged OPH [64,65,66,67]. At the same time, on the basis of the data obtained, as a rule, several important parameters can be estimated: the localization of molecules stabilizing the dimer of the enzyme on its surface, the possible overlap of the active cents of the enzyme, and the affinity of the interaction between the molecules of the “partners” [64,65,66,67,68,69,70,71].

As it was found, various polyelectrolytes can act as such enzyme-stabilizing molecules (Table 2); these molecules themselves do not possess antimicrobial properties, but they can be used in antimicrobial preparations [64,65,68]. However, the most interesting options are those when the molecules involved in the stabilization of the enzyme themselves have antimicrobial action [19,65,66,67,68,70]. These compounds include various so-called antimicrobial polypeptides (AMPs), which are usually of natural origin and can be synthesized by different living organisms. It should be noted that computer models of the structures of various enzyme–polyelectrolyte complexes (EPCs), obtained on the basis of His_6_-OPH and various biopolymers, have shown that, in some cases, there is a “positive” effect of such interactions on the catalytic activity of His_6_-OPH. At the same time, the obtained EPCs had 20–40% greater catalytic efficiency of the enzyme action in hydrolysis reactions, especially at elevated temperatures, compared with soluble His_6_-OPH [64]. 

Due to the known distribution of positively and negatively charged amino acid residues on the surface of the His_6_-OPH dimer [8], polyanion polymers (biodegradable poly(amino acids), such as poly-l-aspartic acid (PLD_50_) and poly-l-glutamic acid (PLE_50_) [64,65,69,71], are preferable as partners in EPCs.

The combination of such EPCs with different antibiotics (ampicillin, gentamicin, kanamycin, rifampicin, and meropenem) [19,64,69] revealed that the presence of the enzyme in a stabilized form helps to reduce the minimum inhibitory concentrations of antibiotics. At the same time, all tested enzyme preparations were equally effective in the hydrolysis not only of AHLs but also of various OPCs. Thus, a prerequisite was created for the production of dual-acting products with both antimicrobial activity and the ability to hydrolytically destruct organophosphorus neurotoxins. Simultaneously, some EPCs had an increased efficiency of catalytic action at a lower pH of the medium compared to the soluble form of the enzyme, which is an important factor for the solubility and antimicrobial action of a number of antibiotics applied with EPCs. 

It was established that between the combinations of studied antibiotics with non-peptide chemistry, which were tested with His_6_-OPH/PLD_50_ or His_6_-OPH/PLE_50_, the greatest positive effect was achieved for β-lactam antibiotics, such as ampicillin and meropenem [19,64]. Such variants provided a clear, successful result of increasing the antibacterial effectiveness of the created combinations of stabilized QQ-enzyme and blockbuster antibiotics. The most unsuccessful combination of the EPCs with an antibiotic was in the case of rifampicin, which caused the inhibition of His_6_-OPH. Moreover, this was shown by studies conducted in silico (“dry experiments”) and in vitro (“wet experiments").

It is interesting to note that the molecular modeling of the interactions of antibiotics with the surface of stabilized His_6_-OPH revealed the binding of antimicrobials both to the area near the active centers of the enzyme subunits and to the area of contact between the dimer subunits. However, the reason for the good result for β-lactam antibiotics was clearly established, which was that the active centers of the enzyme remained accessible for biocatalysis, while the other antibiotics shielded the entry of substrates into the active centers to varying degrees. In the case of meropenem, during catalytic experiments, it turned out that the presence of this antibiotic along with a stabilizing peptide on the surface of the enzyme even contributed to the expansion of the spectrum of the catalytic action of the enzyme itself [68], but the main reason was that all antibiotics were involved in the formation of non-covalent interactions with the His_6_-OPH.

In a recent study—aimed at studying the possibility of combining His_6_-OPH with various AMPs and conducted in silico in order to obtain EPCs with antimicrobial properties and the preservation of enzyme activity [66]—19 different samples of AMPs were used. Various parameters of their interactions with the enzyme (affinity, charge, contact area, etc.) were determined in the obtained computer models. It was shown that both anionic and cationic polypeptides bind to His_6_-OPH with a negligible influence of their charge on these interactions, which significantly differs from the concept of the inter-charge interactions of molecules. The nanocomplexes of His_6_-OPH with indolicidin and temporin A had the best characteristics according to the results of both the molecular docking and in vitro experiments.

Further development of these studies led to the fact that the inclusion of stabilized forms of the enzyme in bacterial cellulose (BC) [68,71] and cryogel of polyvinyl alcohol (PVA-CG) containing this BC in its mass at a concentration of 0.5% [69], thus allowing for the attainment of new samples of antibacterial drugs, which improved the effectiveness of action in relation to the different types of cells of microorganisms. It should be noted that the introduction of BC into the composition of PVA-CG led to significant hardening of the gel structure.

Extremely interesting results were obtained when the stabilized forms of the enzyme, in the form of EPCs, were introduced into the compositions of various fibrous materials formed, including biodegradable (polylactide, polyhydroxybutyrate, poly-ε-caprolactone) and synthetic (viscose and polyester) polymers [70,71]. Moreover, antibiotics of last-resort (polymyxins) [72] and metal nanoparticles (NPs)(Zn and Ta) were used as antimicrobial agents in such composites for comparison regarding the effectiveness of action. These studies have shown that, firstly, various fibrous materials functionalized by EPCs based on His_6_-OPH can be used for the detoxification of OPCs and the hydrolysis of AHLs, since the enzyme retained its hydrolytic activity and acquired stability with the possibility of the materials’ reuse. Secondly, it was found that the materials themselves, in some cases, affect the antimicrobial properties of the created combinations of EPCs and antibiotics or metal nanoparticles. Thirdly, it turned out that Ta nanoparticles clearly act more effectively on a number of microbial cells than the well-known and widely used Zn in various antimicrobial preparations. At the same time, the result with polymyxins was inferior in comparison to the antimicrobial properties of metal nanoparticles. Fourthly, as a result of obtaining the best combination among the studied variants with EPCs, various materials that provided antimicrobial properties turned out to be composite materials with polyhydroxybutyrate and Ta NPs with notably improved antibacterial effects. Generally, these developments have created a certain basis for the further production of dressings with antimicrobial properties as well as materials with protective properties against chemical and biological influences.

## 4. Stabilized and Immobilized His-Tagged OPH in the Hydrolysis of Mycotoxins

Recently, there has been an intense growth of interest in enzymes that are able to catalyze the hydrolysis of mycotoxins synthesized by various microscopic fungi and accumulated in various agricultural raw materials and products [73]. This interest is due to a possible increase in the level of food safety for humans and animals arising from their consumption of food and feeds contaminated with mycotoxins, respectively, due to their detoxification under mild conditions as a result of the action of various enzymatic catalysts [74,75,76]. It is already known today that lactonases may be among such potentially effective destructors [76,77,78], since individual mycotoxins contain lactone structures similar to AHLs in their structures. Since His_6_-OPH is active in relation to AHLs and catalyzes their hydrolysis similarly to well-known lactonases, this determines the interest in the enzyme under discussion as a possible destructor of not only AHLs but also mycotoxins with corresponding chemical structures.

It should be noted that the use of immobilized forms of lactonases is considered promising since they provide high stability during storage and in catalytic treatments of various objects contaminated with mycotoxins [76,78,79].

The use of combinations of enzymes capable of catalyzing the detoxification of mycotoxins of different chemical structures is considered promising and expedient. The idea can be realized through two approaches. Firstly, there are solutions based on the production of recombinant fusion proteins, where enzymes with different substrate specificities of action become partners for such a protein with combined anti-mycotoxin activity [80]. Secondly, there are solutions based on the combined simultaneous use of enzymes for the effective hydrolysis of various mycotoxins, and His_6_-OPH can be successfully used as one of the enzymes in such combinations [20,81] and stabilized as part of a polyelectrolyte complex with poly-l-glutamic acid (PLE_50_), which had previously shown itself well in the hydrolysis of OPCs and AHLs (Table 1 and Table 2). 

**Figure 2:**
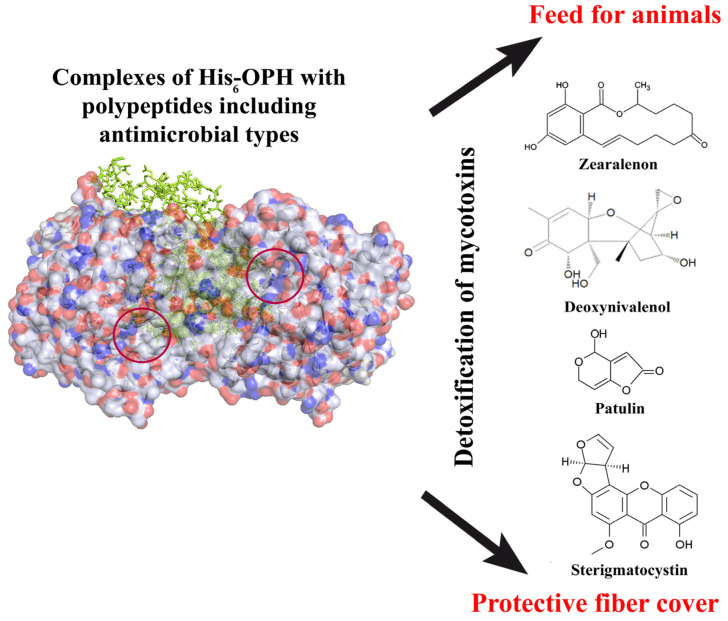
Application of His_6_-OPH in the content of EPC for the degradation of some mycotoxins in the content of animal feed and in the frame of protective fiber cover.

Analogously to OPCs, the interaction of mycotoxins with His_6_-OPH can be computationally modeled [81], thus allowing for the discarding of non-degradable compounds and to pre-select only potential variants at a planning stage. Further, the activity of His_6_-OPH was established in reactions with several mycotoxins: patulin, deoxynivalenol, zearalenone, and sterigmatocystin (Figure 2). It is interesting to note here that sterigmatocystin is a precursor to the biosynthesis of such a highly toxic mycotoxin as aflatoxin B1 [20], and thus, the His_6_-OPH performed properties that were not expected from it.

Interestingly, the combination of His_6_-OPH/PLE_50_ in the destruction reactions of mycotoxins with a similarly obtained EPC based on thermolysin (thermolysin/PLE_50_), which turned out to be able to catalyze the detoxification of ochratoxin A [80], made it possible to join the catalytic potential of both enzymes. A combination of these two EPC variants was used in vivo experiments to detoxify animal feed that was artificially contaminated with several mycotoxins (zearalenone, deoxynivalenol, and ochratoxin A) at concentrations that significantly (up to 300 times) exceeded the permissible concentrations of these toxic substances. Biochemical analysis of animal blood carried out in dynamics during prolonged feeding of Sprague-Dawley rats, as well as the results of the histological and pathomorphological investigations of liver and kidney specimens from euthanized animals showed that enzymatic hydrolysis of mycotoxins proceeded very effectively.

The absorption immobilization of His_6_-OPH in the form of EPCs with PLE_50_, individually as well as in combination with thermolysin/PLE_50_, was made using various fiber materials [20], which were previously applied in the development of composite samples for chemical-biological protection [70]. Further, fiber material samples were contaminated separately with different mycotoxins (zearalenone, sterigmatocystin, and ochratoxin A), and the effectiveness of the detoxification removal of mycotoxins under the action of immobilized enzymes was investigated. It turned out that His_6_-OPH/PLE_50_ completely hydrolyzes zearalenone and sterigmatocystin, whereas thermolysin/PLE_50_ removed ochratoxin A by only two-thirds. An analysis of the catalytic characteristics of His_6_-OPH/PLE_50_ immobilized in fibers in the hydrolytic reactions of mycotoxins showed their improvement by 47 times compared with the soluble enzyme in the reaction with the same substrates. That was a result of the significant stabilization of the enzyme in reactions with mycotoxins due to the immobilization approach used.

Such fiber materials with His_6_-OPH/PLE_50_ (Figure 2) can be considered as a prototype for the creation of protective agents against the action of mycotoxins, which can be used to manufacture special clothing for agricultural workers who have maximum contact with these toxic substances, working with polluted raw materials and feed.

## 5. Comparative Analysis of Biocatalysts Based on Immobilized/Stabilized His_6_-OPH

In analyzing the properties of samples of various immobilized and stabilized biocatalysts based on His_6_-OPH (Table 1 and Table 2), it can be concluded that methods applied to reach the target effect, regardless of the nuances in the implementation of procedures and the chemical reagents used, are characterized by common pros and cons (Table 3). They do not notably differ from those which can be mentioned in relation to many other enzymes immobilized/stabilized by similar approaches. Moreover, in many cases, the methods described here as successive for His_6_-OPH were previously or concurrently applied for other enzymes, always demonstrating some balance between advantages and expected or revealed disadvantages of the stabilizing techniques, used carriers, and their influence on catalytic properties of the obtained biocatalysts.

The use of computer modeling methods appeared effective not only for directed protein engineering of His_6_-OPH (obtaining mutant forms of the enzyme, its fusion proteins, etc.) but also for evaluating and predicting in silico the degree of influence of His_6_-OPH stabilization methods (metal chelating immobilization, polyelectrolyte complexation, bioconjugation with natural molecules, including DNA, etc.) and “partners” selected for the enzyme on the catalytic properties of the biocatalysts obtained. In this regard, it should be continued to expect the active use of computer modeling methods in the future creations of new biocatalytic systems with His_6_-OPH.

Initially, by processing data from different researchers to compile Table 1, the availability of studies to assess the effectiveness of the immobilization options carried out by the authors themselves was analyzed. In a number of cases, the authors have shown that the immobilization efficiency is 100%, while the effectiveness of the procedure assumed that the entire protein (enzyme) that was introduced into the reaction medium was immobilized. As it is known, such results are achieved as a result of preliminary experiments aimed at obtaining highly active biocatalysts, in particular, for the active destruction of organophosphate pesticides in wastewater. However, in a number of studies, the authors of the developments did not set themselves such tasks, conducting fundamental research demonstrating the potential of the possible implementation of certain new technology in relation to His_6_-OPH. In many cases, the authors did not optimize the conditions for the carrying out of their experiments. Sometimes, they immobilized His_6_-OPH, focusing on the positive results obtained in experiments with other enzymes.

Under the effects of immobilization, individual researchers in their works assumed long-term preservation of the catalytic activity of the enzyme and the possibility of its repeated use under periodic conditions [25,26,28,47], and this is another approach to evaluating the effectiveness of the obtained results. In this regard, the positive effect of the immobilization/stabilization of His_6_-OPH on the catalytic activity of the enzyme was chosen as the main criterion confirming the effectiveness of the developments made by different researchers (Table 1 and Table 2). 

It should be emphasized that the catalytic activity was studied against different substrates (methyl parathion [23,25,26,27,28,29,30,37,46,47], paraoxon [31,32,33,34,41,42,43,44,46,47], malathion [36,44], methyl–paraoxon [38], coumaphos [39,40], chlorphyryphos [45], VX [8,29], etc.) under different conditions (mixing and its absence [25,32,44,45], flow reactor [23,82]) at different temperatures and pH values [44]. Since the immobilization/stabilization itself, as it was found [21,26,46,70,80], can cause conformational changes in the active center of His_6_-OPH, affecting the substrate specificity (narrowing or expanding it, giving preference to substrates with a certain chemical structure, in particular, a larger or smaller size of the outgoing group during hydrolysis substrate), then the information in Table 1 and Table 2 as presented may be of interest to obtain a general idea of the scope of research and the results obtained by different methods, but not to compare them with each other. They are too different. In addition, the information provided (Table 1 and Table 2) has shown the variety of solutions proposed over the past 5 years in this area that have a positive practical (remote or already confirmed [8,9,44,45,46,47,83]) result, and the data in Table 3 summarize the various aspects of applying certain approaches and materials to obtain stable forms of His_6_-OPH.

## 6. Conclusions

The use of immobilized His_6_-OPH for the destruction of OPCs, the purification of natural objects, and the development of personal protection systems against the effects of toxic substances, including mycotoxins, is environmentally friendly and, in some cases, economically attractive due to the ease of implementation of the applied methods of enzyme stabilization and possible solutions to existing problems. Approaches are used that are well known and widely used for the immobilization of various enzymes [81], as well as completely new ones developed in recent years to work with different enzyme systems and for special use in studies with His_6_-OPH, since they take into account its characteristics and features. The methods of computer molecular modeling play a huge role in this. Numerous examples have shown that immobilized variants of His_6_-OPH are characterized by increased stability, and the hydrolytic process of destruction of many substrates can be carried out in wider temperature and pH ranges than when using a free form of the enzyme, multiple uses of enzyme biocatalysts are applicable. The variation in methods and carriers for the immobilization of His_6_-OPH makes it possible to create a wide palette of biocatalysts, significantly expanding the boundaries of enzyme use.

## Figures and Tables

**Table 1:** Samples of immobilized and stabilized forms of His_6_-OPH for reactions with OPCs.

Variants of His-Tagged OPH or PTE [Reference]	Immobilization Technique and Carrier	Properties	Application
Use of metalorganic interactions and frameworks			
OpdA@Ni-NTA-VMSN [23]	Ni^2+^-nitrilotriacetic acid (NTA)-modified, virus-like, mesoporous silica nanoparticles	The affine capacity of substrates became higher for the enzyme after its oriented immobilization	Hydrolysis of methyl parathion in a plug-flow reactor
OpdA@MIL-88A [24]	MOF synthesized on the basis of fumaric acid and FeCl_3_	The catalytic activity is 5 times higher as compared to the free form of the enzyme. The enzyme possessed improved organic detergent and solvent tolerance and thermal and storage stability	Degradation of organophosphorus pesticides on grapes and cucumbers
BSA-Cu@CaPs-OPH [25]	A hybrid organic–inorganic, calcium phosphate-containing nanocrystal, based on the use of bovine serum albumin modified by Cu^2+^ ions	Improved multiple usages with up to 56% retainment of activity after 10 working cycles; advanced thermal and pH stability	Hydrolysis of methyl parathion on fruits or vegetables
OPH^6His^/UiO-66-NH_2_ [26]	MOF, synthesized using amino-terephthalic acid and zirconium chloride with further pre-activation by *N*,*N*′-dicyclohexylcarbodiimide, was covalently bonded with the enzyme over the surface via UiO-66-NH_2_	Improved storage stability (up to 60 days), reusability (in 9 working cycles), and 37% improved catalytic activity	Degradation of methyl parathion
OPH^6His^@Tb-BTC [27]	Encapsulation of the enzyme in MOF, formed by terbium nitrate and 1,2,4-benzenetricarboxylic acid (BTC)	Improved storage stability and a 30% increase in activity	Sensing of methyl parathion
OpdA@Co/C@SiO_2_@Ni/C [28]	An enzyme created multiple affine coordination bonds with Co and Ni introduced into carbonized hybrid nanocomposite ZIF-67@ SiO_2_ with a yolk-shell structure	Increased pH, thermal and storage stability, and SDS resistance; long reusability (in 7 working cycles) with 60% of residual activity	Chemo-enzymatic cascade degradation of methyl parathion
Co/MnHF@PTE [29]	An enzyme was involved in the multimetallic nanoflower structure during its formation in a mixture of metal salt solutions and protein: Co-PTE (CoHF@PTE) and Mn-PTE (MnHF@PTE) hybrid	The improved catalytic activity of 4 times due to the use of two metals (Co and Mn) in the frame of the nanoflower-like structure of MOF	Hydrolysis of methyl parathion, VX and soman
OPH@H-Au-TiO_2_ [30]	An enzyme was adsorbed on the hollow-structured nanoparticles, Au-TiO_2_	Increased reusability in the destruction of OPCs and very stable activity due to the synergistic combination of photo- and enzymatic catalysis	Degradation of methyl parathion
OPH@0.8CoZIF [31]	An enzyme was encapsulated to Zn-doped Co-based ZIF via biomimetic mineralization	The enzyme is involved in a cascade of chemical reactions where the 4-aminophenol is the last product	Conversion of methyl parathion
YT-PTE on FE [32]	A mutant enzyme was immobilized by sorption on the Fuller’s Earth (FE)	The temperature and storage stability were improved, but there were no notable changes in the activity of the immobilized enzyme compared to its free form; several bivalent ions (Co, Ni, Cu, Fe, Zn) exhibited significantly higher increases in the activity of the immobilized enzyme	Hydrolysis of paraoxon
Use of fusion proteins and bioconjugations			
[scGFP-arPTE][S^−^] [33]	Fusion proteins, containing green fluorescent protein (GFP) and PTE, were involved in an electrolytic interaction with monomeric S^−^ with further drying and cross-linking to obtain a catalytically active porous film or cross-linking in the presence of cotton fiber to obtain composite textiles	Improved thermal stability and higher catalytic constant (*k*_cat_)	Introduction into cotton fibers for the creation of personalized protective composite material useful for the recyclable hydrolysis of paraoxon
[arPTE][S^+^][S^−^] [34]	Obtainment of bioconjugates through the subsequent introduction of the enzyme into contact with cationic (Ethoquad) and anionic (oxidized IGEPAL) polymer surfactants (S^+^ or S^−^), resulting in the formation of such a structure as corona encapsulating the enzyme	Enhancement of the efficiency of catalytic action by 3 times	Hydrolysis of paraoxon
[arPTE][S^+^][S^−^] –ABC, [arPTE][S^+^][S^−^] –PCL [35]	An enzyme, in the presence of cationic or anionic polymer surfactants (S^+^ or S^−^), was lyophilized with further melting of the obtained powder and used in co-dispersion with curtain polymers (ABC, acrylonitrile butadiene styrene; PCL, polycaprolactone) for 3D-printing on the surface of stainless steel rings	Improved stability of enzyme action	Hydrolysis of paraoxon-ethyl by composite enzyme plastics developed for use in self-decontaminating surfaces
*Po*OPH_M9_-CLEPC [36]	An enzyme containing 10 residues of genetically introduced phenylalanine was conjugated with Pluronic F127 to form a cross-linked enzyme–polymer conjugate (CLEPC)	Increased optimal temperature (50 °C) and pH stability in the range of 7–11	Hydrolysis of malathion
OPH@pID-MSN [37]	An enzyme was covalently immobilized on mesoporous silica nanoparticles (MSNs) coated with a zwitterionic polymer containing short hydrophobic chains, which is a product of the ring-opening reaction between poly (isobutylene-alt-maleic anhydride) and *N*,*N*-dimethylethylenediamine (pID)	Significantly improved stability	Hydrolysis of methyl parathion
Sorption and covalent immobilization on natural carriers			
OPH/PCD [38]	An enzyme was adsorbed onto the microparticles of poly-β-cyclodextrin (PCD) with further freeze-drying	Increased sorption capacity of the substrate and product of the enzymatic hydrolysis; possible regeneration of activity via new enzyme sorption and the self-decontamination of the biocatalyst	Hydrolysis of methyl paraoxon
OpdA-PHB [39]	An enzyme was immobilized on non-porous poly(hydroxyl butyrate) (PHB) microspheres	The carrier increased the sorption of the hydrophobic substrate from the medium	Hydrolysis of coumaphos
SpOpdA-SPS-S [40]	An enzyme was immobilized via the covalent binding of His-tag as SpyTag (Sp) to such fusion proteins as SpyCatcher (SPS)-coated poly(hydroxy alkanoates) (PHAs) spheres (S)	Improved catalytic activity and stability	Hydrolysis of coumaphos
Conjugation using DNA molecules			
QD-DNA-PTE [41]	An enzyme was attached, via the DNA-containing linker, to PEGylated quantum dots (QDs)	Enhanced efficiency and rate of catalytic reaction (*k*_cat_)	Hydrolysis of paraoxon
DNA cage-QD-PTE [42]	Molecules of DNA were conjugated into a cage by His_5_-peptide and modified by quantum dots (QDs) of ZeS with further immobilization of PTE	Enhanced catalytic activity by 12.5 times	Hydrolysis of paraoxon
PTE^pAzF^ -DNA [43]	An enzyme was conjugated to a DNA scaffold modified by dibenzocyclooctyl via a site-specific incorporated azido-group to the enzyme molecule structure	Improved catalytic constants	Hydrolysis of paraoxon
Formation of complexes			
OPT−PIMs [44]	An enzyme (OPT) was involved in the formation of pollen-inspired microparticles (PIMs), prepared based on complexation between CaCO_3_, gelatin, and the enzyme	Improved stability at 50 °C and low pH 4.8 (citric acid/sodium citrate buffer)	Detoxification of pollen contaminated by paraoxon or malathion
His_6_-OPH/PLE_50,_ His_6_-OPH/PLD_50_ [45]	Enzyme was involved in polyelectrolyte complexes with poly-l-glutamic acid (PLE_50_) or poly-l-aspartic acid (PLD_50_)	Increased stability of catalytic action in the soil	Destruction of chlorpyrifos in different types of soil
His_6_OPH/PEG_113_PLE_10_, His_6_OPH/PEG_113_PLE_50_, His_6_OPH/PEG_113_PLE_100_, His_6_OPH/PEG_113_PLD_50_, His_6_OPH/PEG_22_PLE_50_, His_6_OPH/PLE_50_PEG_113_PLE_50_ His_6_OPH/Hydroxyethyl starch His_6_OPH/Succinylated gelatin [46]	An enzyme was involved in polyelectrolyte complexes with PEGylated poly-l-glutamic acid (PLE_10-100_), poly-l-aspartic acid (PLD_50_) of a different polymerization degree, hydroxyethyl starch, or succinylated gelatin	20–40% increased catalytic efficiency of enzyme action	Hydrolysis of methyl parathion and paraoxon
PCL-RHP-OPH [47]	An enzyme was immobilized through electrospinning in poly-ε-caprolactone fibrous mats in the form of a lyophilized complex dissolved in toluene with random heteropolymers (RHPs)	Enhanced stability and reusability (40% residual activity after everyday use for 3 months)	Hydrolysis of methyl parathion and paraoxon

**Table 2:** Stabilized forms of His_6_-tagged OPH developed using antimicrobialagents.

Forms of His_6_-Tagged OPH [Reference]	Method of Enzyme Immobilization	Antimicrobial Agent	Antimicrobial Effect
Polyelectrolyte complexes of His_6_-OPH			
His_6_-OPH/PLD_50_ [64]	The formation of EPC with poly-l-aspartic acid (PLD_50_) in the presence of an antibiotic	Antibiotic is one of the following: ampicillin gentamicin kanamycin rifampicin	The best result for the catalytic activity of His_6_-OPH/PLD_50_ was obtained with the β-lactam antibiotic ampicillin
His_6_-OPH/PLD_50_ [65]	The formation of EPC with poly-l-aspartic acid (PLD_50_), with the further introduction of polymyxin B and emodin	Polymyxin B and inhibitor of quorum sensing	The triple nanoparticles composed of the QS effector, QQ enzyme, and antibiotic demonstrated a significantly improved antimicrobial effect
His_6_-OPH/AMP [66]	The formation of EPC with an antimicrobial peptide (AMP)	AMP is one of following: indolicidin temporin A polymyxin B polymyxin E hepcidin dermcidin bactenecin 2A cecropin A cecropin B kappacin A enkelytin CAP-18	A positive effect was obtained only for the His_6_-OPH/AMP when indolicidin or temporin A was applied as the AMP partner for the enzyme
His_6_-OPH/Bacitracin [67]	The formation of EPC with an antimicrobial peptide, such as bacitracin	Bacitracin	An increase of 3.5–8.5 times the antibiotic effect was in relation to gram-negative bacterial cells and yeasts
Polyelectrolyte complexes of His_6_-OPH in/on various materials			
His_6_-OPH/AMP/BC [68]	The sorption of EPC with an antimicrobial peptide (AMP) onto bacterial cellulose (BC) fiber samples	AMP is one of following: polymyxin B colistin oritavancin dermcidin temporin A indolicidin	A prototype of new dressing material with enhanced antibacterial activity owing to the use of His_6_-OPH in a complex with polymyxin B or colistin for sorption onto BC
His_6_-OPH/PVA-CG/BC [69]	The entrapment of EPC with an antimicrobial agent (β-lactam antibiotic or antimicrobial peptide) into the poly(vinyl alcohol) cryogel (PVA-CG) with inclusions of bacterial cellulose (BC)	The antimicrobial agent is one of the following: meropenem temporin A indolicidin	A combination of His_6_-OPH with meropenem or temporin A before its inclusion into the PVA-CG gave the best catalytic stability and antimicrobial activity
His_6_-OPH/PLE_50_/Polymyxin His6-OPH/PEG-PLE_50_/Me NPs [70]	The sorption of EPC with PEGylated or non-PEGylated poly-l-glutamic acid (PLE_50_) on the fiber materials, containing antibiotic or metal nanoparticles (Me NPs)	Antibiotic is one of the following: polymyxin B polymyxin E metal nanoparticles of Zn or Ta	The prototypes of new fiber materials for chemical-biological protection with improvement by 1.5–2.1 times and 2.9 times the antimicrobial effect and hydrolytic activity against OPC, respectively
His_6_-OPH/PLE_50_/Ta NPs/fiber material [71]	The introduction of EPC with poly-l-glutamic acid (PLE_50_) into the fibrous materials (bacterial cellulose polylactide or nonwoven fiber material containing 70% viscose and 30% polyester) modified by poly-ε-caprolactone or polyhydroxybutyrate and functionalized by tantalum nanoparticles (Ta NPs)	Tantalum nanoparticles	A notable improvement in the antibacterial effect was confirmed in composite materials with polyhydroxybutyrate and Ta NPs.

**Table 3:** General characteristics of the biocatalysts obtained by various methods of His_6_-OPH immobilization.

Type of Stabilization/Immobilization	Main Advantages	Main Disadvantages	References
Metal chelating interactions of His_6_-OPH	Simple procedure realization; Combination with enzyme purification and isolation from cell debris; Stable long-term usage; High enough catalytic activity due to unshielded of active sites for interaction with substrates	Chaotropic agents and pH of the medium can influence metal chelating interactions of the His_6_-tag of the enzyme with a used carrier; the activity of obtained biocatalysts depends on the chelating modifier of the carrier and used metal	[2,16,23,82]
Covalent binding of His_6_-OPH	Stable long-term functioning; possible washings of carriers with immobilized enzymes	Notable (30–40%) decrease in catalytic activity as compared to the native enzyme	[2,37,40]
Involvement of His_6_-OPH in interactions with MOF	Various forms of MOFs can be applied; A possible combination of catalytic and biocatalytic reactions	Steric hindrances; decrease in catalytic activity due to diffusion limitations	[24,26,27,29]
Sorption of His_6_-OPH on carriers	Very simple procedure of biocatalyst obtaining; High enough level of catalytic activity as compared to the original enzyme	Desorption depends on various factors; low enough levels of stabilization	[32,38,39,68,70]
Polyelectrolyte complexation of His_6_-OPH	Simple procedure; High catalytic activity	Possible shielding of active cites of the enzyme; decomplexation with the release of His_6_-OPH	[8,9,33,34,35,45,64,65,66,67,68,70,71]
Involvement of His_6_-OPH in bioconjugation	Simple procedure; High stability of obtained biocatalysts	Possible blocking of active sites; decrease in catalytic activity compared to original enzyme	[33,34,35,43]

## Data Availability

Not applicable.
